# Predicting Leadership Status Through Trait Emotional Intelligence and Cognitive Ability

**DOI:** 10.3390/bs15030345

**Published:** 2025-03-11

**Authors:** Bogdan S. Zadorozhny, K. V. Petrides, Yongtian Cheng, Stephen Cuppello, Dimitri van der Linden

**Affiliations:** 1 Department of Psychology, University College London (UCL), London WC1E 6BT, UK; 2 Thomas International, Marlow SL7 2NL, UK; 3 Department of Psychology, Erasmus University, 3062 PA Rotterdam, The Netherlands

**Keywords:** leadership, trait emotional intelligence, IQ, machine learning (ML), cognitive ability, TEIQue, random forest (RF)

## Abstract

Many interconnected factors have been implicated in the prediction of whether a given individual occupies a managerial role. These include an assortment of demographic variables such as age and gender as well as trait emotional intelligence (trait EI) and cognitive ability. In order to disentangle their respective effects on formal leadership position, the present study compares a traditional linear approach in the form of a logistic regression with the results of a set of supervised machine learning (SML) algorithms. In addition to merely extending beyond linear effects, a series of techniques were incorporated so as to practically apply ML approaches and interpret their results, including feature importance and interactions. The results demonstrated the superior predictive strength of trait EI over cognitive ability, especially of its sociability factor, and supported the predictive utility of the random forest (RF) algorithm in this context. We thereby hope to contribute and support a developing trend of acknowledging the genuine complexity of real-world contexts such as leadership and provide direction for future investigations, including more sophisticated ML approaches.

## 1. Introduction

In the context of the modern workplace, leadership has been defined as the ability of an individual to modify the capacities and motivations of others in order to attain organizational goals (Silva, 2016). This aptitude has been argued to be dependent on a variety of factors, including charisma (Grabo et al., 2017), cognitive ability (Judge et al., 2004), personality traits (de Vries, 2012; Do & Minbashian, 2020), and emotional intelligence (Saha et al., 2023). If an individual lacks the capacity to lead, then rather than motivating their followers to perform, they achieve precisely the opposite result, producing demotivation and burnout (Leary et al., 2013).

In the late 1930s, Lewin and colleagues identified three styles of leadership: namely, autocratic, democratic, and laissez-faire (Ferguson et al., 2006). This classification was further developed by Goleman (2000), inspired by his prior pioneering work on emotional intelligence, with each type of leadership derived from specific emotional intelligence abilities. For instance, authoritative leaders were described as being high on self-esteem and empathy (Drzewiecka & Roczniewska, 2018; Goleman, 2000). Since the turn of the millennium, an enormous number of novel leadership classifications have been developed, but substantial criticisms have been levied both at individual frameworks of leadership styles and at leadership categorization as a whole, from the standpoint of statistical misspecification and imprecise construct definition and measurement (Fischer & Sitkin, 2023).

Outside of the leadership style quagmire, contemporary investigations of leadership can be classified largely into two major strands of research: leadership effectiveness and leadership emergence (Badura et al., 2022; Galvin et al., 2023; Ilies et al., 2004; Judge et al., 2002). Leadership effectiveness has been defined as the ability of the individual to motivate and influence others in order to attain organizational objectives (Amagoh, 2009) and is also linked to the related constructs of leader achievement and performance (Connelly et al., 2000). On the other hand, leadership emergence is a concept that is derived from the encapsulations of leadership that regard it as a dynamic and procedural phenomenon (Fischer et al., 2016). It has been defined as the extent to which an individual is regarded as a leader by others, irrespective of whether one is assigned a leadership role within an organization (Hanna et al., 2020; Judge et al., 2002). In contrast, the formal assignation of a managerial role within an organization provides an objective measure of leadership status (Cuppello et al., 2023). In other words, while leadership emergence primarily describes peer perceptions of leadership, formal leadership assignment usually follows established organizational processes that determine who is selected for leadership roles (DeRue & Ashford, 2010). The distinction between informal perceptions of leadership (i.e., leadership emergence) and formal assignment of leadership positions (i.e., leadership status) is important because organizations rely on a combination of factors, including consideration of the skills possessed by individuals and past experience, when selecting appointees for managerial positions (Yukl, 2010). Therefore, the actual job position that an individual holds at their company or organization can be utilized as a dependent variable, which is in alignment with prior research (Cuppello et al., 2023). Such an operationalization thereby allows for insight into the specific components of differential psychology that predict the actual real-world attainment of leadership roles (Kaiser & Craig, 2011).

An unprecedented confluence of factors are presently contributing to the disruption of the status quo in the world of modern business, including the encroachment of artificial intelligence (Perifanis & Kitsios, 2023), the hybridization of work in the post-COVID environment (Gulliksen et al., 2023), the decentralization of management and the related development of organizational flattening (Evans & Greenway, 2023), the importance of cultural intelligence in increasingly multinational teams (Zhang et al., 2022), and the erosion of team stability due to the rapid reduction in permanent full-time jobs across industry sectors (Bartash, 2024; Evans & Greenway, 2023). Therefore, the examination of management becomes an ever more important endeavor due to the new inherent challenges and expectations posed to leaders by the burgeoning business and technological climate. Notably, all the aforementioned transformational trends share a common thread in that they require emotional attributes to effectively address.

The connection between emotional intelligence (EI) and leadership status is one that had been explored a number of times in the literature, but the relationship obtained between the two constructs has been inconsistent (Kim et al., 2021; Morfaki, 2022; Siegling et al., 2014; Tabassum et al., 2023; Walter et al., 2011). This may be partially ascribable to the wide assortment of EI tests that have been developed and their noninterchangeability (Walter et al., 2011). The dichotomous nature of EI in the face of ability vs. trait emotional intelligence has been extensively established (Petrides, 2011). In brief, the former variety of EI describes it as an aptitude best captured by maximal performance tests, whereas the latter conceives emotional intelligence as an assortment of perceptions, thus bringing the construct closer to the realm of personality, where traits are preponderant (Petrides et al., 2007).

Traits are attributes within the domain of individual differences that are temporally and situationally stable and serve as quantifiable indicators and predictors of individual attitudes, behaviors, choices, and thus life outcomes (Antonakis, 2011). Accordingly, trait EI’s temporal stability has been demonstrated for test–retest durations up to four years (Zadorozhny et al., 2024). In addition, it has been linked to a variety of work-related measures, including transformational leadership (Russ et al., 2023; Schreyer et al., 2023; Siegling et al., 2014), leadership self-efficacy (Villanueva & Sánchez, 2007), leadership status (viz., leader vs. non-leader; Morfaki, 2022; Russ et al., 2023; Siegling et al., 2014), job performance (Doǧru, 2022), job satisfaction (Miao et al., 2016; Petrides & Furnham, 2006; Russ et al., 2023), lower levels of stress (Russ et al., 2023), work engagement (Lestari & Sawitri, 2017), teamwork effectiveness (Farh et al., 2012), and innovation (Winton & Sabol, 2024). In short, trait EI appears to play a salutary role in the career context, as evidenced by its associations with a wide gamut of outcomes interlinked with workplace success, and therefore, career advancement, thus helping to explain its purported connection with leadership.

Trait EI is also significantly correlated with all five of the Big Five personality traits, which are, in turn, especially in the case of conscientiousness and extraversion, linked with job performance (Barrick et al., 2001; Petrides et al., 2010). Consequently, traits were found to play a role in predicting and explaining patterns in leadership overall, including leadership effectiveness and emergence, thereby justifying the assessment of leadership itself through the trait paradigm (Antonakis, 2011; Doornenbal et al., 2022).

Leadership status and cognitive ability have been found to be inconsistently associated, in that the links between the constructs that were reported in prior cross-sectional investigations of leadership were not obtained in longitudinal studies (Daly et al., 2015). Conversely, cognitive ability has been found to be correlated with both leadership effectiveness (0.17) and emergence (0.65; Judge et al., 2004). The connection between role seniority and cognitive ability would appear to be logical, given the reasonable assumption that job complexity increases in line with seniority (Cuppello et al., 2024). Interestingly, although it has been reported that the more intelligent an individual is, the more they are perceived as a leader, this trend was observed to reverse beyond an IQ of 120 (Antonakis et al., 2017; Dipboye, 2018). In combination with the mixed results with respect to the connection between leadership status and cognitive ability, the curvilinearity of this relationship suggests that the connection between leadership and cognitive ability is more complex than was previously supposed, and prescribes the use of more elaborate forms of analysis than simple correlations and linear regressions, which may obscure and distort true effects (Antonakis et al., 2017). Moreover, this finding suggests that cognitive ability alone cannot sufficiently describe leadership status.

Furthermore, the relationship between cognitive ability and trait EI has been found to be inconsistent, ranging from negligible to weakly positive to even negative (Treglown & Furnham, 2023b; Uhrich et al., 2021).

Therefore, while both trait emotional intelligence and cognitive ability appear to play a role in predicting leadership, their impact is too complex to be accurately assessed using conventional statistical techniques. This necessitates the use of modern analytical methods that allow for modeling non-linear relationships, incorporating structural relations within independent variables, and including additional predictors, such as gender, age, education, and employment status (Acton et al., 2019; Badura et al., 2022; Evans & Greenway, 2023). Despite the existence of powerful analytical techniques that have elsewhere been widely adopted—namely, machine learning (ML)—the plurality of studies currently being published in the field of business psychology are still using more traditional statistical methods (Lee et al., 2022). An important limitation of linear models is their inability to adequately model interactions and to handle large and complex datasets (Doornenbal et al., 2022). Additionally, ML offers higher predictive accuracy as compared to traditional linear models, the ability to model nonlinear patterns, and greater adaptability to new data, which enhances research generalizability (Jumin et al., 2020; Pargent et al., 2023; Zhou et al., 2017).

ML can be generally defined as an algorithmic approach that autonomously determines (i.e., ‘learns’) the methods and parameters necessary in order to find the optimal solution to a given problem, without the need for a priori human intervention (Dwyer et al., 2018). ML approaches are generally categorized into unsupervised and supervised ML (SML; Jiang et al., 2020). The distinction between the two is that the former functions with minimal human supervision and is designed to detect patterns in existing data, while SML is designed to learn a function that can predict unidentified dependent data based on a provided sample of input–output pairs and is used for classification and regression tasks (Shetty et al., 2022). Thus, an SML algorithm is able to provide a fitted model which produces an output (i.e., a dependent variable) on the basis of a prediction that is generated by means of a set of one or more features (that is, independent variables or predictors; Nasteski, 2017).

In SML, the dataset is first partitioned into two subsets, namely, the training and the testing datasets, that are usually split in ratios that range from 70:30 to 80:20, in an effort to avoid overfitting (Joseph, 2022). Overfitting is a phenomenon in which a developed model performs perfectly on the training data but then struggles to manage novel data that is dissimilar to those in the training set. Specifically, this occurs because overfitted models simply memorize the entirety of the data rather than learning the patterns underlying them (Ying, 2019). Additionally, by repeatedly partitioning the data, one can simulate replication attempts to enhance reproducibility, through a process referred to as cross-validation (Koul et al., 2018).

The different SML approaches include conventional regressions such as linear and logistic regressions, ensemble models including random forests (RFs) and gradient boosting (GB), along with standalone models including support vector machines (SVMs). The distinction between standalone and ensemble models is that the latter integrate a number of standalone models together through various methods in order to improve prediction accuracy (Roy et al., 2024).

One common type of standalone model is the decision tree, which uses algorithms in order to make choices as to where to partition data, forming branches, and splitting the data into increasingly more granular segments (McCarthy et al., 2019). A random forest (RF) is a type of algorithm that is composed of individual decision trees, which is proficient at handling non-linear relationships, interactions, correlated predictors, and data heterogeneity (Janitza et al., 2016). As RFs construct numerous decision trees and generate aggregate trees by averaging across them, they consequently reduce overfit (Jiang et al., 2020).

Gradient boosting (GB) is another algorithm that iteratively combines weaker standalone models into a stronger ensemble, thus increasing accuracy over time (Natekin & Knoll, 2013). When decision trees are used as the base, this produces a resultant algorithm that is referred to as a gradient-boosted tree (Krauss et al., 2017; Vaulet et al., 2022).

SVMs treat each predictor as a dimension and look for the optimal hyperplane (essentially, a straight line in two-dimensional space, a two-dimensional plane in three-dimensional space, and so forth) that best separates the classes within the data (Noble, 2006). SVMs can utilize different types of kernels which are families of functions that are used in order to detect patterns in the data through maximizing separation between variables (Bi et al., 2019). These include linear-, polynomial-, radial-, and sigmoid-type kernels (Elen et al., 2022).

All ML algorithms include hyperparameters, which are non-data-related attributes that define how a model performs. Prior to initiating prediction on the testing dataset, it is a best practice to ‘tune’ the hyperparameters, which is the process of finding the optimal values for these attributes, in an effort to attain maximal model performance (Agrawal, 2021).

### Present Study

Given the evidence that leadership status is partially predicted by trait emotional intelligence and cognitive ability as well as an assortment of demographic factors, and considering the limitations of traditional statistical approaches, this study was designed to examine the predictive influence of trait emotional intelligence and cognitive ability in the real-world business context by utilizing a binary logistic regression as the base comparison model, followed by SML methodologies. All study participants completed the instruments as part of their employment and were classified as either managerial or non-managerial, which served as the operationalization of leadership status in this study, consistent with prior research (Cuppello et al., 2023).

The primary aim of this study was to predict leadership status on the basis of trait emotional intelligence and cognitive ability as operationalized by the Trait Emotional Intelligence Questionnaire (TEIQue; Petrides, 2009) and the General Intelligence Assessment (GIA; Dann, 2015). We assessed to what extent trait EI (Russ et al., 2023; Siegling et al., 2014) and cognitive ability (Cuppello et al., 2024) were able to predict leadership position through distinguishing leaders from non-leaders, while incorporating the following other predictors:

1. Age, given the results of prior studies and the role of job experience and age preference for managerial appointments (Chaturvedi et al., 2012; Cuppello et al., 2024);
2. Gender, considering the well-documented gender gap in leadership attainment (Carli & Eagly, 2011);
3. Educational attainment, respective of the role of leadership training in educational programs and the requirement of educational credentials for many managerial positions (Hanna et al., 2020);
4. Employment status, i.e., full-time or part-time employment, due to differences in responsibilities between the two modalities of management (McDonald et al., 2009).

It was expected that, overall, trait EI would be a stronger predictor of leadership status than cognitive ability, controlling for the other factors, in accordance with prior findings (Siegling et al., 2014).

## 2. Methods

### 2.1. Participants

There were 6247 participants (37.3% female, 62.7% male) assessed as part of occupation-related activities who were employed at various companies and across different job sectors. Employees who completed both the cognitive ability and the trait emotional intelligence assessments were included in the sample. The mean age of the sample was 38.0 years (age range: 18 to 69 years; SD = 10.0 years). Data were collected on the highest level of education attained by participants, with 37.2% having completed high school or lower, 41.7% holding bachelor’s degrees, 4.0% holding an MBA, 16.2% holding a different master’s degree, and 0.99% possessing doctorates; 35.1% of the sample (*n* = 2190) were non-managers and 64.9% (*n* = 4057) were managers; 5.9% of the sample were employed part-time and 94.1% of the sample were employed full-time. All participants were located in the United Kingdom and completed testing in English.

### 2.2. Measures

The Trait Emotional Intelligence Questionnaire (TEIQue; Petrides, 2009) consists of 153 items that are rated on a 7-point Likert scale, ranging from 1 (completely disagree) to 7 (strongly agree). The assessment comprises 15 facets at its lowest level of interpretation, 13 of which then load onto four intermediate factors (viz., well-being, self-control, emotionality, and sociability), while the remaining two facets load directly onto the apex-level global trait EI score.

The General Intelligence Assessment (GIA; Cuppello et al., 2024; Dann, 2015) was used to assess cognitive ability. The GIA is a multi-axial multiple-choice test that was developed specifically for the career context and measures both general cognitive ability and trainability, with perceptual speed, verbal reasoning, number speed and accuracy, word meaning, and spatial visualization as the comprising subscales (Dann, 2015). Test scores are adjusted for guessing and are then converted to T-scores by means of comparison to norm group data to generate a percentile rank that compares the respondent to the general working population in the relevant comparison group. In the present instance, the comparison norm group was designed to be representative of the general UK working population. As the GIA test is founded in item generation theory, there is not a static number of questions (Dann, 2015).

Both instruments used in this study are commercially marketed and distributed worldwide by Thomas International. Cronbach’s alphas are presented in Table A1.

### 2.3. Procedure

The assessments were administered electronically to a sample of individuals recruited from a variety of companies within the United Kingdom by a British psychometric test publisher. All participants were provided with feedback on their scores and consented to the analysis of their anonymized test data.

The data were collected by a psychometric test publisher. At the time of data collection, participants were informed that their anonymized results may be used for research purposes. Prior to the transmission of data for analysis, all data were fully and irreversibly anonymized, so that the identity of individuals could not be subsequently recovered.

### 2.4. Data Cleaning

Prior to data analysis, the data were prepared and cleaned. The original dataset consisted of *n* = 74,588 individuals who had completed the TEIQue assessment. First, participants who had not completed the GIA assessment in addition to the TEIQue were removed from the dataset, which led to the exclusion of 52,286 cases (*n* = 22,302 remaining). Next, individuals who did not report their gender (*n* = 231) were excluded (*n* = 22,071 remaining). Following this, individuals who were students, self-employed, or did not report their employment status were also removed (*n* = 6558 excluded, *n* = 15,513 remaining). Individuals who did not report their managerial level or reported it as ‘other’ were excluded next (*n* = 1016 excluded, *n* = 14,497 remaining). Similarly, individuals for whom educational data were unavailable were excluded (*n* = 3461 excluded, *n* = 11,036 remaining). Individuals for whom age data were not available were removed following this (*n* = 281 excluded, *n* = 10,755 remaining).

When the data were inspected on the basis of TEIQue facet scores (which are expected to range from 1 to 7), 13 cases were found for which facet scores were reported as above 7. Therefore, the decision was made to recalculate all TEIQue scores (including facet, factor, and global scores) on the basis of the available raw item scores on the TEIQue questionnaire. As part of this process, one case was deleted due to having zeroes in raw item questionnaire responses (*n* = 10,754 remaining). Five additional cases were deleted due to their age being reported as under 18 (*n* = 10,749 remaining). It was decided to assess only UK-based participants, which resulted in a dataset of *n* = 6247 individuals.

### 2.5. Data Analysis Plan

Statistical analyses were carried out using R v. 4.4.1 (R Core Team, 2021) and RStudio v. 2024.04.2 (R Studio Team, 2020), including the use of the ‘caret’ package (Kuhn, 2008). The advantage of ‘caret’ is that it includes a vast number of machine learning (ML) algorithms, enables simple hyperparameter tuning, and allows for cross-validation.

The dataset was randomly split using an 80/20 ratio prior to initiating analyses, with 80% utilized as the training dataset for model fitting, with the remaining 20% retained as the testing dataset for the subsequent evaluation of model performance. A random seed was set for reproducibility. The threshold was set to 0.5 for classification, as is standard for binary classification tasks. Ten-fold cross-validation was used, in accordance with established best practices (Malakouti, 2023).

A binary logistic regression was carried out in order to serve as a control model for model performance comparison. The independent variables (predictors) were age, gender, educational attainment, employment status (part-time vs. full-time), the General Intelligence Assessment (GIA) factor scores (viz., perceptual speed, verbal reasoning, number speed and accuracy, word meaning, and spatial visualization), and the TEIQue factor scores. The binary dependent variable was leadership status (manager vs. non-manager). The adequacy of the model fit was assessed by means of the ROC (receiver operating characteristic) AUC (area under the curve) values. Guidelines for ROC AUC thresholds were selected from Hosmer et al. (2013) and were as follows: 0.70 < AUC < 0.80, 0.80 < AUC < 0.90, AUC > 0.90, indicating acceptable, excellent, and superior discrimination, respectively.

The first step in the ML process was to tune the hyperparameters for the selected models on the designated training portion of the data. In this study, rather than performing a grid search, the hyperparameters were tuned automatically, which has been demonstrated to be a superior approach (Hutter et al., 2015). Categorical predictors were dummy-coded, utilizing one-hot encoding. Ultimately, the following ML algorithms were used: random forest (RF) via the ‘ranger’ function (caret method: ‘ranger’), support vector machines (SVM) using a radial kernel (‘svmRadial’), and gradient boosting using boosted trees (‘xgbTree’), for each of which automatic hyperparameter tuning was performed. The models were evaluated by means of comparing their performance on the test data, through the use of balanced accuracy, as well as sensitivity, specificity, precision, Cohen’s kappa, and AUC values.

Once the best ML model was selected, the relative importance of its predictors was assessed. This was carried out by means of perturbation, as developed by Breiman (2001), and in accordance with Doornenbal et al. (2022), wherein predictor importance is determined through the adding of random change to a given predictor, whilst holding the other variables constant, followed by the evaluation of resultant change in the outcome variable. Specifically, the higher the increase in prediction error that is produced when a variable is permuted, the greater importance that predictor holds within the model (Doornenbal et al., 2022; Schneider & Brühl, 2023). This was carried out using the ‘caretEnsemble’ package (Deane-Mayer, 2024). Specifically, the change in mean absolute error (MAE) was calculated respectively for each permuted variable. This value was then normalized by comparing it to the change in MAE when the outcome predictions were permuted. A score of 1.00 indicates that permutations of a given variable had an impact equivalent to permuting the outcome variable directly.

With the help of the ‘vivid’ package (Inglis et al., 2022), we calculated interactions between all predictors in the testing dataset utilizing the selected best ML model by means of H-statistics (Friedman & Popescu, 2008), which is a measure that utilizes partial dependence to evaluate the strength of the effect of pairwise interactions between predictors on the outcome variable. This was then visualized through the construction of a heatmap and a network plot. Individual interactions were further investigated by means of grouped scatterplots, which were produced using the testing dataset.

## 3. Results

### 3.1. Logistic Regression

Table 1 shows the results of the binary logistic regression analysis. This model was designed to predict the binary outcome of leadership status (0 for non-managers and 1 for managers) on the basis of the trait emotional intelligence and cognitive ability factor scores, while controlling for age, gender, educational attainment, and employment status.

The model’s goodness-of-fit was assessed using a likelihood ratio, which produced a significant result, *χ*^2^(16) = 1304.20, *p* < 0.001, thereby refuting the null hypothesis. Furthermore, the model’s pseudo-R^2^ values indicated that approximately 20.0% (McFadden) to 31.5% (Nagelkerke) of the variability in leadership status was accounted for by the model in comparison to the null model.

Three of the four trait EI factors, viz., self-control, emotionality, and sociability, significantly predicted leadership status. Specifically, the probability of being in a management position increased 2.73x (95% CI [2.36, 3.17]) for every one unit increase in sociability and 1.13x (95% CI [1.01, 1.27]) in self-control. Conversely, the odds of being a leader decreased by 1.29x (95% CI [0.61, 0.82]) for every one unit increase in emotionality.

Notably, none of the General Intelligence Assessment (GIA) factor scores significantly predicted leadership status.

Other significant positive predictors of leadership status were gender (being male rather than female) (1.22x, 95% CI [1.04, 1.42]), age (1.09x, 95% CI [1.09, 1.10]), possessing a non-MBA master’s degree (1.47x, 95% CI [1.20, 1.79]), and possessing an MBA specifically (3.19x, 95% CI [1.89, 5.80]).

On the other hand, part-time employment status reduced leadership status three-fold (0.33x, 95% CI [0.25, 0.44]), as did possessing a maximum educational attainment that was at the high-school level or lower (0.67x, 95% CI [0.58, 0.78]).

**Figure 1:**
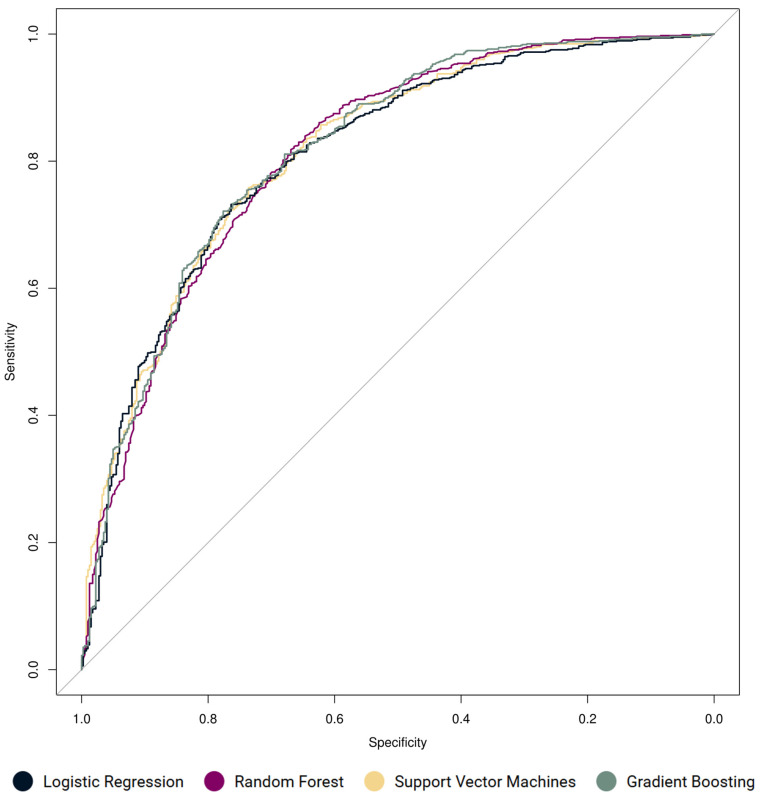
Comparison of ROC curves for the constructed models.

The AUC (area under the curve) for the ROC curve was calculated to estimate the adequacy of the logistic regression and was found to be 0.811, suggesting an excellent level of discrimination (see Figure 1). The balanced accuracy of the model was 0.710.

### 3.2. Machine Learning Algorithm Comparison

Three different ML algorithms were used in the present study to predict the binary outcome of leadership status from the study predictors. The performance of the algorithms was assessed first on the training data and was initially compared to the performance of the linear logistic regression. The hyperparameters for each algorithm were also trained during this step and the optimal parameter values were as follows: random forest (RF, ranger): number of randomly selected predictors (mtry) = 6, splitting rule = extratrees; support vector machines with radial kernel (SVM, svmRadial): sigma = 0.0481502, cost = 0.25; gradient boosting (GB, xgbTree): number of boosting iterations (nrounds) = 100, maximum tree depth (max_depth) = 1, shrinkage (eta) = 0.3, minimum loss reduction (gamma) = 0, subsample ratio of columns (colsample_bytree) = 0.6, minimum sum of instance weight (min_child_weight) = 1, and subsample percentage = 0.8888889.

With respect to the training stage, the best two models performed approximately equally: SVM (balanced accuracy = 0.717 and AUC = 0.811) and GB (balanced accuracy = 0.718 and AUC = 0.818). See Figure 1 for a visualization of AUC performance and Table 2 for the full comparison of model performance. All models showed an excellent level of discrimination in accordance with the selected threshold guidelines.

At the testing stage, the best performing model in terms of balanced accuracy was the RF model (0.736), followed by the GB (0.728) and the SVM (0.723), with the logistic regression in last place (0.710). The RF model also performed the best with respect to sensitivity, specificity, precision, and Cohen’s kappa, but not AUC. The performance of all models was better on the testing data than on the training data, indicating that overfitting did not occur.

### 3.3. Analysis of the Selected Machine Learning Model

**Figure 2:**
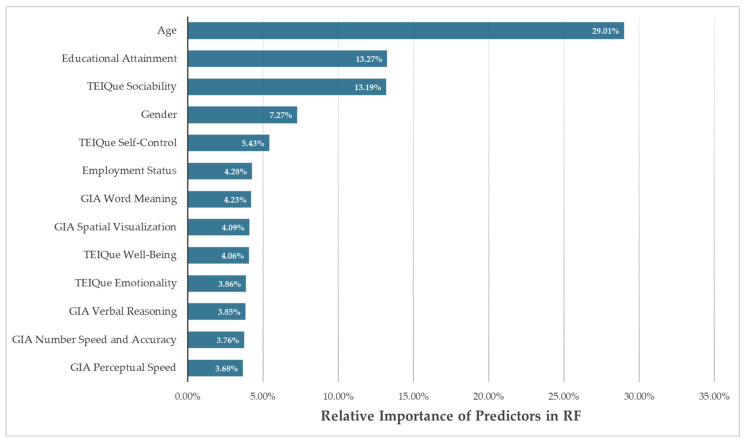
Relative variable importance in the RF model.

The predictor variables in the RF model were permuted in order to estimate their predictive importance. See Figure 2 for a visualization of the permutation results. Age had the greatest impact on prediction accuracy (29.01%), followed by educational attainment (13.27%), trait EI sociability (13.19%), gender (7.27%), and trait EI self-control (5.43%), with the remainder of the variables each demonstrating a relative importance below 5%.

**Figure 3:**
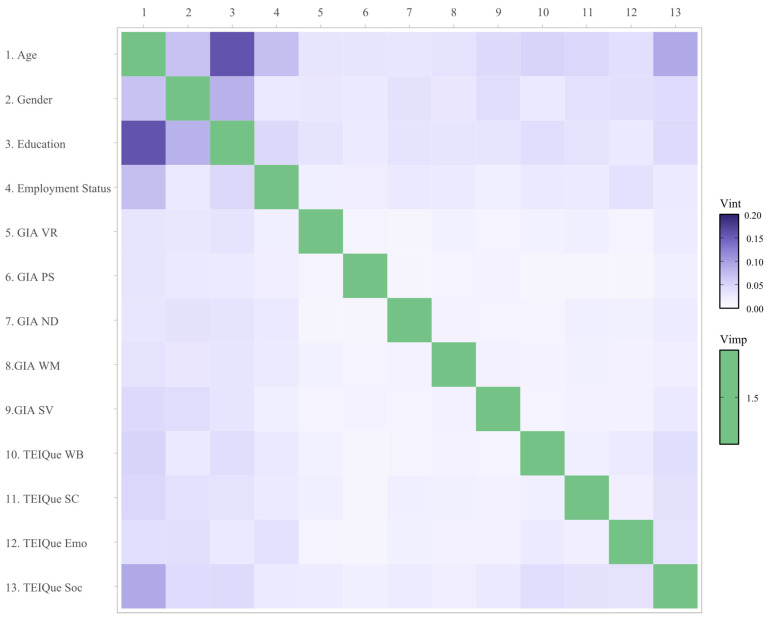
Heatmap of variable interactions in the RF model. Note: The factors of the General Intelligence Assessment (GIA) are VR = verbal reasoning; PS = perceptual speed; ND = number speed and accuracy; WM = word meaning; SV = spatial visualization. The factors of the Trait Emotional Intelligence Questionnaire (TEIQue) are WB = well-being; SC = self-control; Emo = emotionality; Soc = sociability.

**Figure 4:**
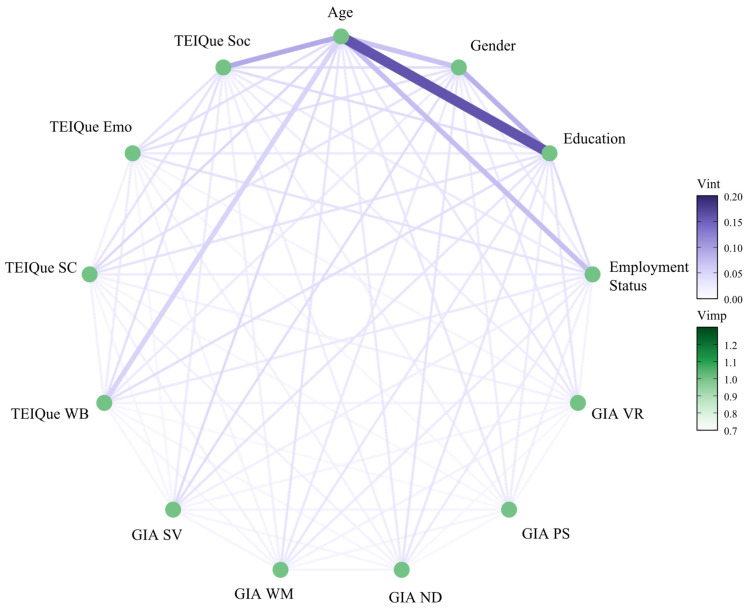
Network plot of variable interactions in the RF model. Note: The factors of the General Intelligence Assessment (GIA) are VR = verbal reasoning; PS = perceptual speed; ND = number speed and accuracy; WM = word meaning; SV = spatial visualization. The factors of the Trait Emotional Intelligence Questionnaire (TEIQue) are WB = well-being; SC = self-control; Emo = emotionality; Soc = sociability.

Next, the presence of interactive effects on the outcome was assessed amongst all predictors. A heatmap plot visualizing the importance of interactive effects on leadership status is presented in Figure 3, with a comprehensive table of interactive effects presented in Table A2. The same data are also shown in an alternative format, using a network plot, in Figure 4. Interaction effects were calculated as the proportion attributable to their interaction out of the overall combined effect of the two variables, as a percentage (unnormalized pairwise interaction strength).

**Figure 5:**
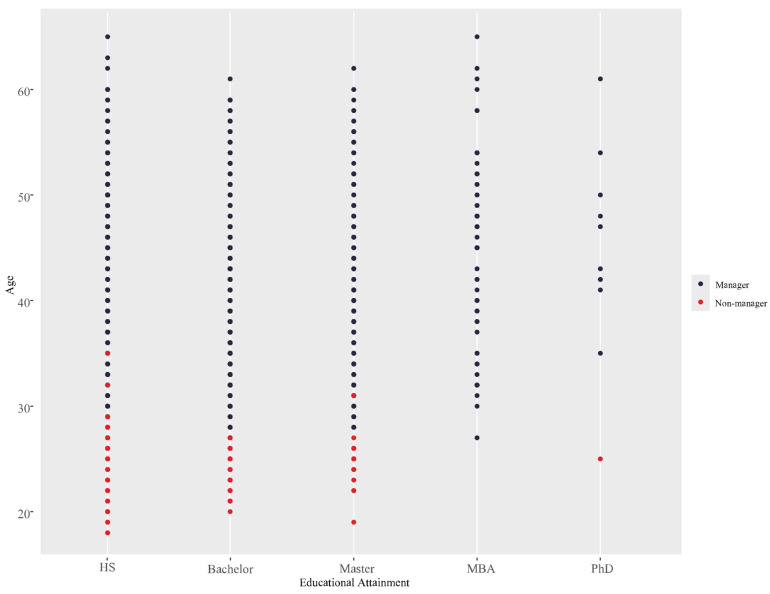
Interaction between age and educational attainment using the RF model. Note: The levels of educational attainment are presented on the x-axis, representing the highest level of educational attainment, in ascending order: high school (HS), bachelor’s degree, master’s degree, Master of Business Administration (MBA), and doctorate (PhD).

**Figure 6:**
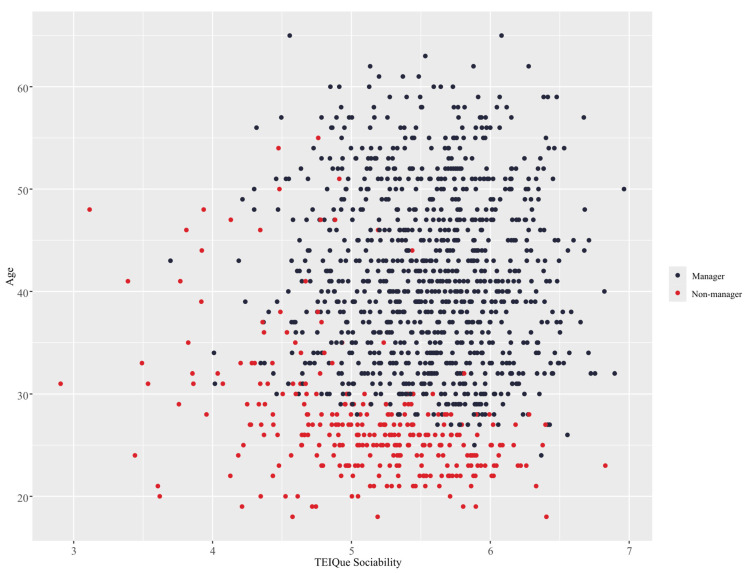
Interaction between age and trait EI sociability using the RF model.

**Figure 7:**
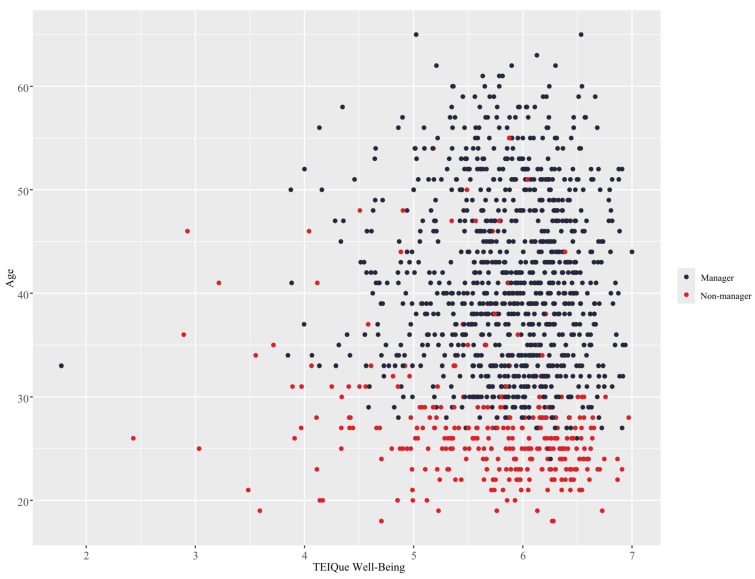
Interaction between age and trait EI well-being using the RF model.

**Figure 8:**
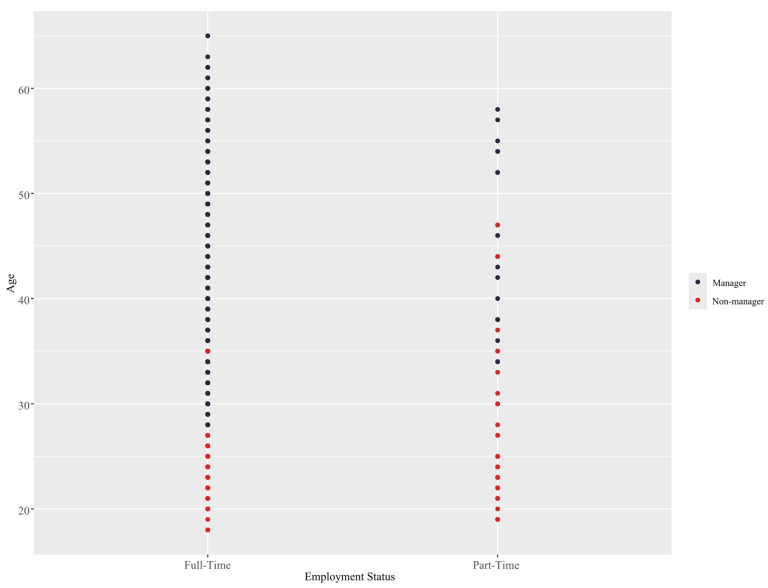
Interaction between age and employment status using the RF model.

The strongest interactive effects on predicting leadership status were between age and the other predictors, in respective order of strength: educational attainment (15.96%), the trait EI factor of sociability (9.16%), employment status (7.29%), gender (6.99%), and the trait EI factor of well-being (5.27%). The only other interactive effect that exceeded 5.00% was amongst educational attainment and gender (8.40%). Selected interactions are illustrated below, in Figure 5, Figure 6, Figure 7 and Figure 8.

## 4. Discussion

### 4.1. The Isolated Effects of Trait Emotional Intelligence and Cognitive Ability on Leadership Status

The results of the logistic regression supported the hypothesis that trait emotional intelligence (EI) would be a stronger predictor of leadership status than cognitive ability (controlling for age, gender, educational attainment, and employment status), in line with prior findings in the field (Birknerová et al., 2021; Siegling et al., 2014; Treglown & Furnham, 2023a). Indeed, the present results indicated that none of the five factors of the General Intelligence Assessment (GIA) were a significant predictor of leadership, controlling for age, gender, educational attainment, and employment status. This was contrary to the findings of a prior study that utilized the GIA to predict management level while controlling for age and gender, which had found all five subtests of the GIA to be significant predictors (Cuppello et al., 2024). However, as that study did not incorporate trait EI as a predictor, and in lieu of the aforementioned relationship between trait EI and leadership, it can be hypothesized that the relationship between cognitive ability and leadership status is largely subsumed within trait EI’s relationship with the latter. In other words, hidden relationships amongst trait EI and cognitive ability that are normally occluded on the global level of analysis, cause cognitive ability to no longer be significantly related to leadership status when both it and trait EI are included as predictors within a model.

With respect to the trait EI factors, three predicted leadership status in the logistic regression, namely, sociability, self-control, and emotionality, in respective order of strength. The trait EI factor of sociability reflects the common core of its constituent facets (i.e., assertiveness, emotion management, and social awareness), therefore emphasizing the interpersonal, social aspect of trait emotional intelligence (Petrides, 2009). This aligns with extant research, the results of which have shown that interpersonal effectiveness increases in importance in more senior leadership positions (Zaccaro, 2007). The superior predictive strength of sociability is likely also partially attributable to its aforementioned components of assertiveness and social awareness, which have both been linked to leadership (Gough, 1990). Although it has been argued that the relationship between assertiveness and leadership is curvilinear, it is likely that the two facets can balance each other out when both are at high levels, producing an optimal state of equilibrium with respect to leadership (Ames & Flynn, 2007). Excessively high scores on emotionality indicate an inclination toward overindulgence in emotional expression, which may explain this facet’s negative relationship with leadership status. This aligns with findings that control over one’s emotions and their expression is a key requirement for leadership (Petrides, 2009; Rajah et al., 2011). The negative relationship between TEIQue emotionality and leadership status aligned with prior findings, as did the non-significant predictive role of TEIQue well-being (Treglown & Furnham, 2023a).

With respect to other independent variables, age significantly predicted leadership status, in alignment with hypotheses. This corresponds with the established and well-known practice of favoring older individuals for career promotion and can also be explained by the fact that both tenure and years of work experience are tied to age (Cuppello et al., 2024; Paoletti et al., 2020). This aligned with prior findings that indicated that age constitutes a formal criterion for organizational promotion (Ng et al., 2005). Another potentially related contributing factor is the trend in individuals retiring at increasingly older ages (Fry & Braga, 2023).

Also, as predicted, gender had an effect on leadership status, with males being 22% more likely to occupy a leadership role, which corresponded with extant literature (Carli & Eagly, 2011).

The highest level of educational attainment was likewise a significant predictor of leadership status, with greater attainment linked with a higher likelihood of occupying a leadership position, apart from doctoral-level graduates who were underrepresented in the study sample. The most interesting relationship was the significantly greater probability of MBA graduates of occupying leadership roles as opposed to all other levels of education, including other master’s degrees. In fact, possessing an MBA degree was the strongest predictor of leadership status of all. There are multiple potential explanations for the strength of this relationship: individuals aspiring for management positions are likelier to complete MBAs (especially relative to other master’s degrees), MBAs may enhance employability through skill development and networking, and finally, employers may prefer or require applicants for leadership positions to hold MBAs (Baruch & Peiperl, 2000; Mihail & Kloutsiniotis, 2014).

On the other hand, part-time employment was a negative predictor of leadership status, which was likely due to the rarity of that modality in leadership positions (Funk & Warning, 2024).

### 4.2. Venturing Beyond Linear Effects

To explore whether the relationships amongst trait emotional intelligence, cognitive ability, and leadership status were more complex than linear models can represent, a series of supervised machine learning algorithms were deployed. Going beyond linear modeling has the advantage of enabling the construction of predictive models that correspond with modern best practices and recommendations in the field of leadership research (Doornenbal et al., 2022).

The model that was utilized as the baseline for comparison in the present study was a logistic regression, the results of which were described above. It is a type of generalized linear model, which implies that linearity is assumed (between the predictors and the logit of the outcome variable) and that the results that it produces are strictly a sum of its parameters (Stoltzfus, 2011). In other words, simple logistic regressions are strictly additive and are thus unable to account for interactions. Moreover, multiple linear regressions that do enable the analysis of interactions require the interactive terms to be explicitly defined a priori while adding spurious interactions can potentially increase both Type I and Type II error (Afshartous & Preston, 2011). An important limitation of logistic regressions and linear models generally is their requirement of limited multicollinearity amongst predictor variables (Vatcheva et al., 2016).

Due to this limitation of linear models and inspired by the capabilities offered by machine learning (ML) approaches, we proceeded to create predictive models, of which two were ensemble and one was a standalone ML model. The two ensemble models generated were the random forest (RF) and the gradient-boosted (GB) tree, while the standalone was a support vector machine (SVM) with a radial kernel.

According to all metrics, the performance of all three models in predicting leadership status was superior to the logistic regression on the testing data. The comparison of the predictive performance of the models revealed that ensemble models performed best, followed by the standalone SVM model, while both types of models outperformed the logistic regression. The superior performance of the ensemble models over the SVM and the logistic regression indicates the existence of a likely non-linear relationship between the predictors and the criterion of leadership status (Schneider & Brühl, 2023). Additionally, the finding of a (slight) performance edge of RF over SVM concurs with extant findings on the comparative classification performance of the two algorithms (Díaz-Uriarte & Alvarez de Andrés, 2006; Statnikov & Aliferis, 2007). Notably, past studies that investigated leadership using ML techniques likewise endorsed RF-based approaches (Doornenbal et al., 2022; Gruda et al., 2021; Lee et al., 2022).

Although the RF demonstrated a performance advantage in terms of its balanced accuracy, sensitivity, and Cohen’s kappa, and was ahead of the rest in terms of its specificity and precision, its AUC (area under the curve) value was lower than that of both the SVM and the GB model, and was only marginally higher than the baseline model itself. However, as can be seen in Figure 1, the logistic regression performs clearly worse in the critical regions. This issue is covered in the literature and precluded us from using ROC as the main comparative metric (Lobo et al., 2008). Simultaneously, some authors have also cautioned against the use of Cohen’s kappa as a measure of model performance and therefore we did not use it as the principal method of comparison either (Delgado & Tibau, 2019). Rather, we relied on balanced accuracy.

### 4.3. Digging Deeper: Permutation Analysis and Interactive Effects

The permutations that were conducted on the predictors in the RF model enabled us to rank their predictive importance. As with the logistic regression results described previously, age was again revealed to be a significant predictor of leadership status, but in the RF model results, it was also the most important. This implies that the logistic regression underestimated the extent to which age predicts leadership. The machine learning analysis told the same story as the linear model: trait EI sociability, gender, trait EI self-control, and educational attainment remained the strongest predictors of leadership status, preserving their relative order of importance. Conversely, employment status modality appeared to be far less important than it was in the logistic regression, which may be related to the small (5.9%) proportion of part-time employees in our sample. Finally, trait EI emotionality, which had an unexpected negative relationship with leadership status in the logistic regression, has only a negligible relative importance in the RF model, perhaps indicating the previously obtained negative relationship to be spurious. This is supported by the fact that prior studies that examined the connection between leadership (as defined as occupying a leadership role) and this trait EI factor reported this relationship to be in the expected positive direction (Benson et al., 2013; Morfaki, 2022). Conversely, said studies only examined linear effects and were conducted on small sample sizes, suggesting additional investigation is warranted.

The other major advantage of ML approaches is that rather than resigning ourselves to regarding each predictor in isolation, it is possible to inspect their interplay. The strength of the most powerful predictor, age, is further elaborated through its interactions with the other variables in predicting the overall leadership outcome. Of these, the strongest is the interaction between age and education, which is illustrated in Figure 5. As can be seen, individuals who completed a high school diploma as their highest level of educational attainment were predicted to become leaders at an older age than others, while individuals who completed MBAs were predicted to obtain leadership at the youngest age relative to the others, with those who completed bachelor’s and other master’s degrees somewhere in between the two groups.

There were insufficient data to make a solid determination for individuals with PhDs due to their underrepresentation in the sample. Researchers have suggested that educational attainment provides a link between cognitive ability and later career success, while individuals with higher levels of cognitive ability are significantly less likely to drop out of education early (Daly et al., 2015). Therefore, it is possible that the predictor of educational attainment is partially accounting for some of the differences in cognitive ability. Simultaneously, the relationship between MBA completion and leadership status accords with the linear effects outlined above.

Interactive effects were also observed between age and the trait EI factor of sociability, as shown in Figure 6. The RF model predicted that older individuals are generally more likely to be in leadership positions. However, individuals who scored lower than 5 on sociability were predicted not to hold leadership positions, even in later age. It was also shown that individuals under the age of 30 were only predicted to occupy leadership positions if they exhibited higher than average (>5) levels of sociability. Trait EI tends to increase with age, which explains why younger individuals with high trait EI are at a particular advantage (Budler et al., 2022). A plausible explanation could be that of a generational cohort effect. Specifically, some meta-analytic evidence exists to support the theory that while levels of trait EI for individuals remain largely stable, younger generations may possess lower levels of trait EI than prior generations (Khan et al., 2021). This thereby may give rise to a situation where individuals who possess relatively higher levels of EI reap not only the baseline benefits from possessing high levels of trait EI but are even further advantaged from being more competitive relative to their peer group. This is further supported by the fact that the construct’s longitudinal stability has been demonstrated for extended multi-year periods (Zadorozhny et al., 2024).

The finding that trait EI sociability has a significant impact on leadership status is also aligned with prior research that found that sociability was positively related to more senior management levels (Treglown & Furnham, 2023a). However, the results of that study did not indicate that the trait EI factor of well-being also had a relationship with management level, while in the present study, a similar pattern was demonstrated for the trait EI factor of well-being, as illustrated in Figure 7. This difference in findings can be attributable to the superior predictive capability of the RF model or the interactions between age and the trait EI factors.

The interaction between age and employment status is likely due to a fundamental difference between part-time and full-time work modalities, wherein individuals who are employed part-time have restricted access to career development opportunities, training, and professional support (Anne Bardoel et al., 2007). This is especially significant given modern shifts in labor trends wherein an increasing proportion of the working population is employed part-time (van Doorn & van Vliet, 2024). With that said, managers do not typically work part-time (Nesbit, 2006), but there are signs that this is also starting to change (Karlshaus, 2020). On the other hand, there is also a distinction between low-skill and high-skill part-time jobs as well as between involuntary and voluntary part-time employment. The former type of positions (i.e., low-skill and involuntary) are far likelier to be occupied by younger people, such as students, while the latter type of positions are likely attained through seniority, wherein the part-time status of the job constitutes a benefit rather than a limitation (Fagan et al., 2014; Ibáñez, 2011).

Taken together, the results of the logistic regression and the ML approaches supported the hypothesis that trait EI would predict leadership status more strongly than cognitive ability. Indeed, none of the cognitive ability factors were found to be significant predictors. However, it is notable that the most powerful predictors were neither trait EI nor cognitive ability factors but rather demographic variables, specifically, age, gender, and educational attainment. Conducting the analyses on the factorial level also revealed that instead of trait EI predicting leadership status holistically, the constituent trait EI factor of sociability is a far stronger predictor than the other three trait EI factors.

### 4.4. Limitations and Future Directions

The present study had several limitations. While individual demographics were covered to a satisfactory level in the data, with the notable exception of tenure, there was limited information available with respect to other features, such as the type of work being performed. Given that leadership is argued to encompass aspects beyond the individual, including group and organizational factors, other previously unconsidered predictors that may influence how individuals come to occupy leadership positions include organizational size, organizational lifecycle stage, team size, and external perceptions of leadership, such as charisma (Badura et al., 2022; Gardner et al., 2024; Shamir & Howell, 1999). The dataset was not representative of the general population, most significantly as it consisted of more managers than non-managers. In accordance with extant literature, further insight into the nature of the interactions could be afforded through the use of Shapley Additive Explanation (SHAP) dependence plots (Schneider & Brühl, 2023). Additional insights may also be revealed through the use of synthetic data that could enable the assessment of predictive accuracy on a dataset of a size that would be extremely cumbersome to recruit (Hong et al., 2024).

In the present study, the ROC curves intersected and the differences between AUC values were rather small, casting potential doubt on the distinctiveness of the predictive accuracy of the different models. However, there are certain approaches that enable enhanced comparison when ROC curves intersect and it is plausible that such analyses may yield additional insight into comparative model performance (Gigliarano et al., 2014). Alternatively, predictive performance can be enhanced further through the use of the ‘SuperLearner’ package that utilizes an advanced ML approach known as ensemble machine learning, wherein several standalone ensemble models (sub-models) are combined in order to develop an average model that may have a predictive accuracy that is greater than that of the comprising sub-models (Polley et al., 2024; Zhao et al., 2023).

Additionally, in consideration of the cross-cultural stability of trait EI (Petrides et al., 2007) and the presumed cross-cultural differences in leadership structures (Roth, 2022), it would be worthwhile to conduct a multinational investigation. Such research could also clarify the nature of the relationship between cultural intelligence and trait EI, as the results of past investigations have been mixed (Alon et al., 2023; Ang & van Dyne, 2008; Crowne, 2009).

Future investigations could examine the effect of the present predictors, including trait EI and cognitive ability on leadership emergence, i.e., the extent to which individuals are regarded as leaders. This could be accomplished by means of an operationalization such as the Conger–Kanungo leadership scale (Conger & Kanungo, 1994) or the General Leadership Impression Questionnaire (GLI; Cook et al., 2021; Cronshaw & Lord, 1987). Additionally, future research could examine how various organizational contexts and selection processes could moderate the relationships that were presently found.

### 4.5. Conclusions

This study investigated to what extent trait EI and cognitive ability factors could predict which employees occupy leadership positions. After analyzing a logistic regression model, we constructed and evaluated three different predictive ML models. To the best of our knowledge, this is the first paper that predicted leadership status using trait EI and cognitive ability factors through the use of ML methods. The findings suggest that things appear to be rather more complicated than the clean and stark pictures of the world that generalized linear models can create. In short, everything is complicated. The real-world relationships amongst variables hardly resemble cleanly separable straight lines and are rather more akin to a Gordian knot. ML may be the blade that can slice it open, but merely cutting the knot does not unravel its mysteries. There is clearly much more to be accomplished.

## Figures and Tables

**Table 1:** Logistic regression results predicting leadership status.

Variable	B	SE	OR ^1^	95% CI ^1^	*p*-Value
Age	0.09	0.004	1.09	1.09, 1.10	<0.001
Gender					
Female	—	—	—	—	—
Male	0.19	0.078	1.22	1.04, 1.42	0.012
Educational attainment					
Bachelor	—	—	—	—	—
High school or lower	−0.40	0.076	0.67	0.58, 0.78	<0.001
Other master’s degree	0.38	0.102	1.47	1.20, 1.79	<0.001
MBA	1.20	0.285	3.19	1.89, 5.80	<0.001
Doctorate	0.01	0.355	1.01	0.51, 2.08	>0.90
Employment status					
Full-time	—	—	—	—	—
Part-time	−1.10	0.150	0.33	0.25, 0.44	<0.001
GIA Verbal Reasoning	−0.01	0.005	0.99	0.98, 1.00	0.20
GIA Perceptual Speed	0.01	0.006	1.01	1.00, 1.02	0.08
GIA Number Speed and Accuracy	0.01	0.007	1.01	1.00, 1.02	0.20
GIA Word Meaning	0.01	0.008	1.01	0.99, 1.02	0.40
GIA Spatial Visualization	−0.01	0.008	0.99	0.98, 1.01	0.30
TEIQue Well-Being	−0.11	0.074	0.90	0.78, 1.04	0.14
TEIQue Self-Control	0.13	0.059	1.13	1.01, 1.27	0.032
TEIQue Emotionality	−0.34	0.073	0.71	0.61, 0.82	<0.001
TEIQue Sociability	1.00	0.075	2.73	2.36, 3.17	<0.001

^1^ Refers to the Odds Ratio, and the 95% CI thereof.

**Table 2:** Comparison of machine learning algorithm results on the testing data.

Model	Balanced Accuracy	AUC	Sensitivity	Specificity	Precision	Cohen’s Kappa
Training Data						
Logistic Regression (Baseline)	0.699	0.792	0.857	0.541	0.770	0.417
Random Forest (RF)	0.702	0.792	0.871	0.532	0.770	0.426
Support Vector Machines with Radial Kernel (SVM)	0.717	0.811	0.872	0.561	0.781	0.454
Gradient Boosting (GB)	0.718	0.818	0.862	0.575	0.785	0.454
Testing Data						
Logistic Regression (Baseline)	0.710	0.811	0.875	0.545	0.802	0.442
Random Forest (RF)	0.736	0.814	0.889	0.582	0.818	0.495
Support Vector Machines with Radial Kernel (SVM)	0.723	0.817	0.882	0.565	0.810	0.470
Gradient Boosting (GB)	0.728	0.818	0.876	0.580	0.815	0.476

## Data Availability

The data used in the present study are proprietary and therefore cannot be made accessible.

## Appendix A

**Table A1:** Descriptive statistics, reliabilities, and correlations amongst the study variables.

Variable	*M*	*SD*	1	2	3	4	5	6	7	8	9	10	11	12	13	14
1. Age	37.93	10.12	-													
2. Gender	0.63	0.48	0.13 **	-												
3. Educational attainment	0.90	0.88	0.12 **	−0.05 **	-											
4. Employment status	0.94	0.24	0.08 **	0.16 **	−0.03 **	-										
5. Leadership status	0.65	0.48	0.40 **	0.15 **	0.15 **	0.14 **	-									
6. GIA VR	39.88	8.68	−0.15 **	−0.04 **	0.04 **	0.00	−0.04 **	(0.70)								
7. GIA PS	42.79	6.39	−0.14 **	0.03 *	0.05 **	0.02	−0.02	0.47 **	(0.70)							
8. GIA ND	14.69	6.00	−0.11 **	0.21 **	0.10 **	0.02	0.02	0.42 **	0.41 **	(0.71)						
9. GIA WM	30.19	5.35	0.12 **	0.01	0.15 **	0.02	0.09 **	0.50 **	0.42 **	0.36 **	(0.71)					
10. GIA SV	10.14	5.10	0.02	0.15 **	0.06 **	0.05 **	0.03 *	0.30 **	0.34 **	0.39 **	0.32 **	(0.74)				
11. TEIQue WB	5.83	0.63	0.05 **	−0.04 **	0.06 **	−0.01	0.09 **	−0.04 **	−0.02	−0.03 *	−0.05 **	−0.04 **	(0.72)			
12. TEIQue SC	5.33	0.73	0.17 **	0.15 **	0.05 **	0.03 *	0.15 **	−0.09 **	−0.04 **	−0.05 **	−0.08 **	−0.00	0.53 **	(0.79)		
13. TEIQue Emo	5.62	0.65	0.05 **	−0.18 **	0.04 **	−0.04 **	0.04 **	−0.04 **	−0.02	−0.11 **	−0.03 *	−0.05 **	0.60 **	0.47 **	(0.75)	
14. TEIQue Soc	5.46	0.60	0.13 **	0.13 **	0.03 *	0.07 **	0.24 **	0.01	−0.02	0.03 *	0.02	−0.00	0.56 **	0.42 **	0.50 **	(0.77)

Note: *N* = 6247; *M* = mean; *SD* = standard deviation. The internal consistencies (Cronbach’s alphas) are reported on the diagonal. Dummy codes: gender, 0 = female, 1 = male; educational attainment, 0 = high school or lower, 1 = bachelor’s degree, 2 = master’s degree (other), 3 = MBA, 4 = doctorate; employment status, 0 = part-time, 1 = full-time; leadership status, 0 = non-manager, 1 = manager. The factors of the General Intelligence Assessment (GIA) are VR = verbal reasoning; PS = perceptual speed; ND = number speed and accuracy; WM = word meaning; SV = spatial visualization. The factors of the Trait Emotional Intelligence Questionnaire (TEIQue) are WB = well-being; SC = self-control; Emo = emotionality; Soc = sociability. * indicates *p* < 0.05. ** indicates *p* < 0.01.

**Table A2:** Interactions amongst the study variables.

Variable	1	2	3	4	5	6	7	8	9	10	11	12	13
1. Age	-												
2. Gender	0.07	-											
3. Educational attainment	0.16	0.08	-										
4. Employment status	0.07	0.03	0.04	-									
5. GIA Verbal Reasoning	0.03	0.03	0.04	0.02	-								
6. GIA Perceptual Speed	0.03	0.03	0.03	0.02	0.01	-							
7. GIA Number Speed and Accuracy	0.03	0.04	0.04	0.03	0.01	0.01	-						
8. GIA Word Meaning	0.04	0.03	0.03	0.03	0.02	0.01	0.01	-					
9. GIA Spatial Visualization	0.04	0.04	0.03	0.02	0.01	0.01	0.01	0.02	-				
10. TEIQue Well-Being	0.05	0.03	0.04	0.03	0.02	0.01	0.01	0.01	0.01	-			
11. TEIQue Self-Control	0.04	0.04	0.04	0.03	0.02	0.01	0.02	0.02	0.02	0.02	-		
12. TEIQue Emotionality	0.04	0.04	0.03	0.04	0.01	0.01	0.02	0.02	0.02	0.03	0.02	-	
13. TEIQue Sociability	0.09	0.04	0.05	0.03	0.03	0.02	0.03	0.02	0.03	0.04	0.04	0.04	-
